# Research advances in exercise management for frail older adults

**DOI:** 10.3389/fpubh.2026.1763583

**Published:** 2026-01-29

**Authors:** Caini Liu, Lan Lu, Wei Wei

**Affiliations:** 1 Nursing College, Guangxi Medical University, Nanning, China; 2 Department of Nursing, First Affiliated Hospital of Guangxi Medical University, Nanning, China

**Keywords:** assessment of aging, frail older people, exercise and health management, research progress, tool

## Abstract

As global aging accelerates, there is a dramatic shift in the structure of world health. Frailty marks the entry into aging and becomes an important factor affecting the health of older adults. In order to reduce the harm of infirmity to the physical health of the older adults, international interventions for infirmity have been continuously developed, and exercise management has been widely studied as an effective intervention strategy in geriatric infirmity, but the domestic research on infirmity started late and has been less studied. This paper reviews the definition and clinical manifestations of frailty in the older adults, types of exercise interventions and assessment of exercise effects, and research progress in health management theory, with a view to providing reference for the development of exercise programs for frail older adults.

## Introduction

**Figure 1:**
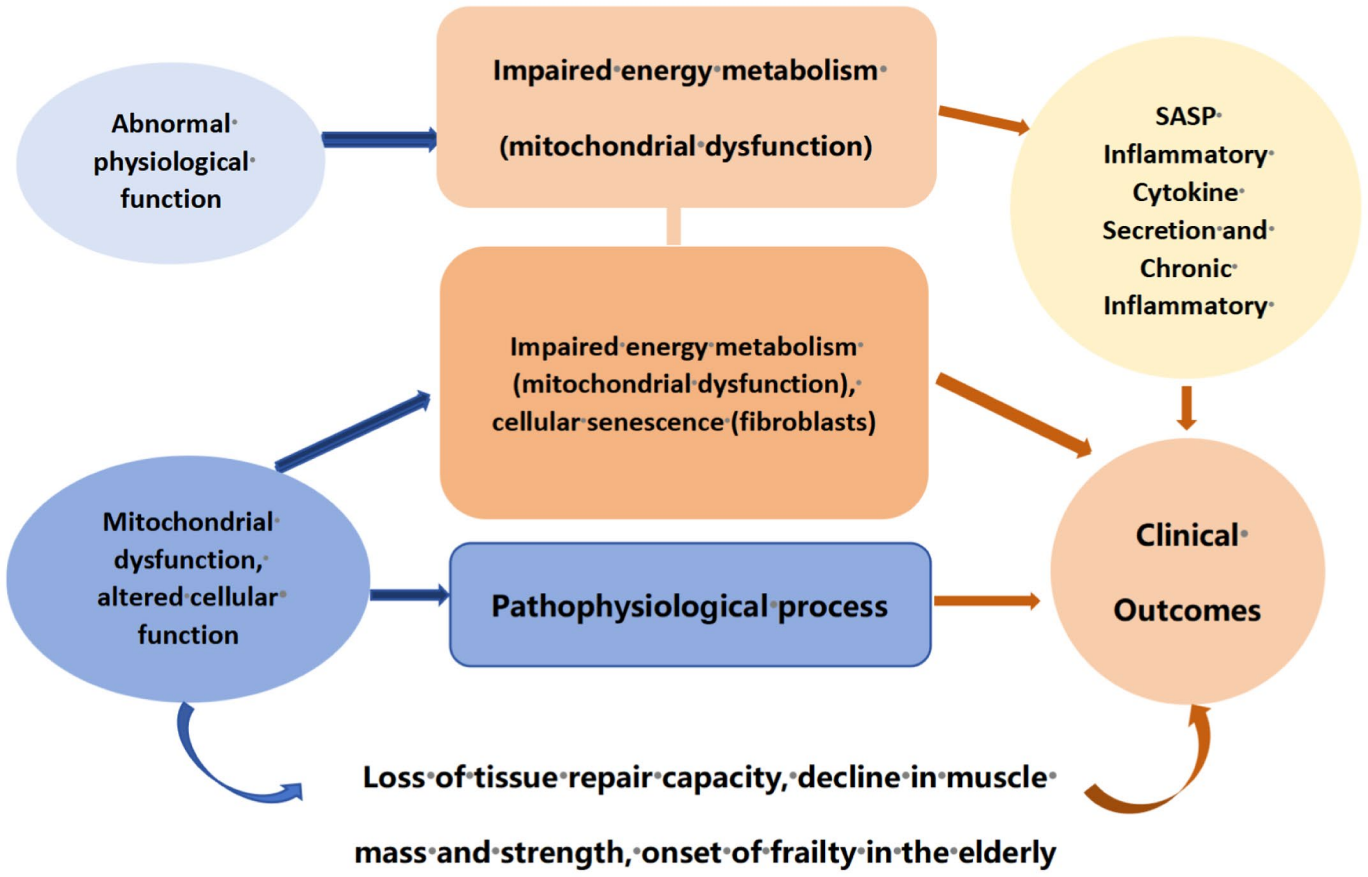
Mechanism diagram of frailty in the older adults.

Frailty is a syndrome with multiple symptoms, often manifested as a decline in the functions of various organs caused by muscle loss, nutritional deficiency, hormonal changes and increased inflammation, thereby reducing the body’s stress tolerance (1, 2). The mechanism of frailty involves multi-system, multi-level pathophysiological changes, with the general mechanism depicted in Figure 1. Frailty is usually manifested as weight loss, fatigue and reduced physical activity, etc. Although frailty is not a disease, it is closely related to an increased risk of falls, disability, hospitalization, institutionalization and eventual death. Along with the accelerated aging of the global population, it has become a major public health problem. At present, the number of older adults patients with frailty is gradually increasing, and the prevalence of frailty is on the rise. Studies show that in some populations, approximately 16. 1% of the older adults can be classified as frailty, while 58. 1% of the older adults can be regarded as in the pre-frailty stage (3). With the occurrence of frailty, the increase of endogenous (such as diseases) and exogenous (such as social and cultural factors, environment) stressors related to frailty also increases the risk of adverse outcomes related to frailty. Given the increasing incidence of frailty and its related health risks, including higher hospitalization rates and mortality rates, it is crucial to adopt effective intervention strategies, and exercise management is one of the most effective intervention strategies.

Exercise is one of the main ways to manage frailty in old age. It can not only maintain various bodily functions and keep muscle strength, but also alleviate negative emotions related to frailty, such as depression and anxiety, thereby improving mental health. Through research, it has been found that exercise can reduce the incidence of frailty among people aged 65 to 74. Strengthening physical exercise is beneficial to improving the frailty state of the older adults with sarcopenia and enhancing their muscle endurance (4). Studies have confirmed that continuous exercise can significantly improve the cardiopulmonary endurance and physical function of patients with heart failure, and have proposed that exercise is an important early warning of physical frailty in the older adults (5). Some studies have shown that exercise can effectively manage frailty. Adopting personalized exercise plans for different individuals can achieve better exercise results, have a more significant effect on improving physical functions, and even completely reverse the frailty state in old age, becoming an important part of comprehensive care for the older adults (6). Exercise for older adults frail patients should be multi-dimensional and comprehensive. Compared with a single form of exercise, the combined intervention of multiple types of exercise can achieve better results (7). Research shows that after multi-component combined exercise intervention for the older adults, multiple indicators such as their muscle strength, balance ability and gait speed can be significantly improved, and the incidence of falls has also decreased. The physical fitness and overall quality of life of the older adults have been significantly enhanced (8). Therefore, exercise management plays an important role in the care of older adults patients with frailty, which can effectively improve the physical function and quality of life of the older adults and reduce the risk of frailty.The evaluation of the effect of exercise intervention is crucial for understanding the effectiveness of improving health outcomes in older adults frail patients, especially among vulnerable groups such as the older adults and those with chronic diseases. Nowadays, there are various assessment scales for the effects of exercise in the international community. Through these scientific assessment tools, it is possible to effectively identify the various functional states, exercise capabilities and potential health risks of patients, thereby providing a basis for formulating personalized exercise intervention plans for older adults patients. In recent years, chronic diseases have become one of the important factors contributing to frailty in many older adults people. There have been a large number of studies on the management of chronic diseases internationally, and various health management theories have been proposed. Many health management theories have been widely applied in the management of various chronic diseases and achieved good results. This article reviews the types of exercise intervention for older adults frailty patients at home and abroad in recent years. The theory of exercise effect evaluation and health management aims to provide healthcare professionals with insights for optimizing the care of frail older adults people through targeted exercise programs.

## Types of exercise management

Exercise is an important management approach to improve the frailty condition of the older adults. Some current research results show that exercise plays a positive role in reducing muscle mass loss in the older adults, maintaining their physical functions and mental health, and can effectively improve the physical health level and mental health. Exercise management mainly combines different types of exercise, including aerobic exercise, strength training, and balance and coordination training, etc. It formulates personalized and effective exercise plans based on the characteristics of the older adults to achieve the effect of maintaining the muscle strength of patients and improving their frailty state. Moreover, according to relevant studies, regular resistance training and aerobic exercise for older adults patients can effectively enhance their muscle mass and strength, and also improve various functions of the body (9, 10). Among them, resistance training can promote the synthesis of muscle protein, inhibit protein degradation, and alleviate the development process of sarcopenia (11). Different types of sports focus on different physical functions. Therefore, it is particularly important to formulate targeted exercise management plans based on the needs of the older adults, Table 1 presents training programs tailored to different levels of frailty.

**Table 1:** Prescription form for decline exercise.

Frailty grading	Type of exercise	Frequency	Intensity	Time (minutes/session)	Progress principle	Core security monitoring and precautions
Pre-frail	Aerobic exercise	3 to 5 times per week (74)	Moderate intensity (RPE 5–6/10) (75, 76); or 50–70% of maximum heart rate (74).	20–40	Start with 20 min per session, increasing by 5 min every 1–2 weeks until reaching the target duration. Begin at a low-to-moderate intensity and gradually increase as you adapt (74).	Conduct a pre-exercise assessment of cardiovascular and physical function risks (74). Monitor for discomfort symptoms during exercise (such as dizziness, chest pain, or excessive shortness of breath). 3. Emphasize the importance of warm-up before exercise and cool-down afterward (74).
	Strength training	At least twice a week, with at least one day between sessions (74)	Moderate intensity (e.g., 50–80% of 1RM) (75); or perform the concentric contraction at maximum speed (power training) (75).	—	Begin with 1–2 sets of 8–12 repetitions each. First increase the number of repetitions, then add resistance or sets (74)	Focus on proper form and avoid holding your breath. Adjust or stop if you experience joint pain.
	Balance training	2–3 times per week	Low to moderate, with training conducted under safe and controlled conditions.	10–15	From supported (e.g., leaning against a wall) to unsupported, from static to dynamic, gradually reducing the support surface (77)	Must be performed with stable support or under supervision to prevent falls.
	Flexibility training	Before and after each workout (74)	Stretch until you feel a slight tension, without pain.	5–10	Gradually increase the range of motion and duration of the stretch.	Avoid rebound stretching; exercise caution with individuals experiencing severe joint mobility restrictions.
Mild–moderate frail	Aerobic exercise	3–5 times per week	Low to moderate intensity (RPE 3–5/10) - 4-10 min (76, 77); starting at 40–50% of maximum heart rate (74).	0–30 min, can be accumulated in intervals (e.g., 10 min per session, 2–3 times daily).	Progress slowly, prioritizing increased exercise duration (no more than 5–10% weekly increase) before considering intensity.	Exercise risk assessment is mandatory; may require supervised initiation (74). Monitor intensity using a combination of heart rate, RPE, and talk test (ability to carry on a simple conversation during exercise). Closely monitor fatigue levels and fall risk.
	Strength training	2–3 times per week	Low to moderate intensity (40–60% of 1RM or using resistance bands) - 5-10 reps	—	Start with 1 set of 4–8 repetitions per set. First ensure proper form and full execution before increasing repetitions, and only then consider adding resistance (78).	Prioritize functional strength training (such as sit-up exercises). Avoid excessive fatigue and ensure adequate rest between training sessions.
	Balance training	2–3 times per week, can be combined with other training sessions	Low intensity, ensuring safety (77, 78).	5–10	Progress from seated balance to standing balance with support from a hand-held object (78).	Under the close supervision of a professional or caregiver.
	Flexibility training	Before and after each daily activity or event	Gentle, slow	5–10	The primary goal is to maintain existing joint range of motion, with gradual attempts at improvement (77).	Avoid putting pressure on painful joints.
Severe frail	Aerobic exercise	Based on tolerance, engage in short bursts of activity multiple times daily.	Very low intensity (RPE 2–3/10), ensuring no noticeable shortness of breath occurs (77, 78).	5 to 10 min each time, multiple times daily	With the goal of reducing sedentary time and increasing daily activity frequency, has the amount of time spent on activities increased each day	Must be conducted under the supervision of a rehabilitation therapist or healthcare professional with personalized assessment (77, 78). Monitor blood oxygen saturation, heart rate, and subjective fatigue levels. Combined with nutritional support (77, 78).
	Strength training	2–3 times per week, or as needed during daily activities (77, 78)	Very low intensity, using body weight or resistance bands with minimal resistance, or performing only gravity-defying movements (77, 78).	—	Use successful completion of the movement as the benchmark for success. Do not focus on increasing resistance; instead, try adding 1–2 additional repetitions.	Monitor pain responses and adjust immediately if any discomfort arises. Prevent contractures and pressure ulcers.
	Balance training	Integrate into daily care and transfers	From passive to active assistance (77, 78)	Irregularly	Begin with passive activities, encouraging patients to participate as actively as possible.	Conducted entirely under supervision and protection, with strict fall prevention measures in place (77, 78).
	Flexibility training	1–2 times daily	Passive or assisted active movement (77, 78)	5–10	Maintain the joint’s current range of motion to prevent contractures (77).	Move gently to avoid injury.

### Aerobic exercise

Aerobic exercise is a kind of physical activity that mainly provides energy through aerobic metabolism and is carried out under the condition of sufficient oxygen supply. It is characterized by low intensity, rhythm and long duration. Aerobic exercise delays and improves the frailty symptoms of the older adults through various physiological effects. Specifically, on the one hand, aerobic exercise improves the cardiopulmonary function of the older adults by enhancing the oxygen exchange efficiency of alveoli, increasing the oxygen-carrying capacity of blood, and strengthening the contractility of the myocardium. On the other hand, it can reduce the occurrence of chronic inflammation, decrease oxidative damage, and resist aging. At the same time, it can also enhance muscle metabolism, increase insulin sensitivity, control blood sugar, and reduce the occurrence of cardiovascular diseases and metabolic syndrome. Current research shows that aerobic exercise can promote the older adults population to maintain a better physical condition and living state by enhancing mitochondrial function, increasing energy metabolism in muscles and autophagy, etc. (12, 13). At the same time, aerobic exercise also plays a role in neural regulation. Continuous aerobic exercise can promote the secretion of dopamine and endorphins, effectively relieve anxiety and depression, and promote mental health. In addition, persisting in aerobic exercise can also reduce the risk of disease in the older adults. Research has found that aerobic exercise has a greater effect on people with high genetic risk and can offset part of the genetic risk (14).

There are many ways of aerobic exercise, such as brisk walking, jogging, swimming, cycling, etc. Due to the special nature of the older adults population, appropriate exercises should be selected based on their characteristics (15). For older adults people who can walk independently or are in the early stage of frailty, they can engage in activities such as marching, brisk walking, jogging, swimming, mountain climbing, aerobics, Baduanjin, Tai Chi, and square dancing (16). For older adults people who need assistive devices to walk, it is recommended to do seated stepping, pedaling or hand-cranked bike exercises. To enhance the coordination of movement and cognitive functions, etc., the older adults are encouraged to engage in dual-task training, such as expanding the chest, patting the legs and punching while stepping. Exercise patterns can be determined based on personal preferences, accessibility, cognitive levels, chronic diseases, and musculoskeletal disorders, among other factors. According to a review, the form of aerobic exercise suitable for the older adults should be combined with individual ability and interest to improve the compliance and effectiveness of the exercise (17).

Frail older adults people who engage in moderate and regular aerobic endurance exercises within their own limits can enhance their aerobic capacity, fatigue resistance and muscle endurance, thereby improving their frailty. Expert consensus recommendation: The exercise intensity is suggested to start from 30% to 40% of the maximum heart rate and gradually increase to 70% to 75%. However, when heart rate is used as an indicator for observing exercise intensity, the impact of some drugs on heart rate should be considered. The intensity of exercise can also be evaluated using the Borg Subjective Fatigue Scale of 6 to 20. For frail older adults people, it is recommended that 11 (relaxed) to 13 (slightly tired) be the optimal intensity, which is manifested as slight sweating of the body and continuous and intermittent communication with people around (18). Exercise duration and frequency: It is recommended that frail older adults people engage in aerobic endurance training 5 to 7 times a week, starting with 5 to 10 minutes each time and gradually increasing to 15 to 30 minutes (19). Although aerobic exercise has a significant intervention effect on frailty, it poses certain safety risks for older adults frailty patients. Personalized exercise plans need to be formulated based on the patients' own conditions.

### Strength training

Strength training is one of the exercises that use external resistance (such as dumbbells, equipment, etc.) or one’s own body weight as the load to increase muscle strength, expand muscle circumference or shape muscle form to improve muscle loss, enhance activity endurance and increase bone density. It is one of the key exercise methods for preventing and alleviating frailty. Strength training mainly stimulates the growth of muscle fibers through anaerobic metabolism and the repetition of multiple sets of movements, aiming to increase muscle strength and endurance. Compared with aerobic exercise, strength training focuses more on muscle training rather than energy consumption, and it places a higher load on the body than aerobic exercise. Appropriate strength training can significantly improve the muscle function and quality of life of the older adults, reduce the incidence of falls, enhance the ability of daily living activities, and delay aging (19). In addition, strength training can improve bone health and muscle metabolic function, and reduce the probability of falls and injuries (20). Meanwhile, strength training is beneficial for maintaining normal blood sugar sensitivity, reducing the incidence of chronic diseases such as cardiovascular diseases, helping the older adults gain self-respect, reducing anxiety and depression, and improving mental health (21, 22). However, strength training is an exercise that imposes a load on the body and has certain requirements for the physical condition of the older adults. It also carries certain risks. Therefore, before engaging in strength training, it is necessary to assess the physical condition of the older adults and choose a suitable method to do moderate exercise.

Strength training comes in various types and can mainly be classified into: traditional resistance training (such as barbell/dumbbell free weights, resistance bands), and functional training (focusing on enhancing the physical abilities of the older adults in daily activities, such as balance, coordination, and flexibility, mainly using their own body weight and simple equipment). And Blood Flow Restriction Training (BFRT), etc. Traditional resistance training: Strength training usually carried out with free weights and equipment, common ones include bench presses, squats, deadlifts and resistance band training, etc. Through muscle exercise, it maintains muscle strength and enhances muscle endurance, suitable for most older adults people (23). Functional training includes gait, balance, coordination and flexibility training, etc. Such training is carried out using self-weight or simple equipment, focusing on exercising the older adult’s ability to complete various activities necessary for daily life, which is beneficial for the older adults to maintain their ability to live independently and take care of themselves. Blood flow restriction training is one of the emerging training methods in recent years. By applying pressure to the proximal part of the limb and restricting venous blood return while ensuring arterial blood supply, it can promote muscle growth under a relatively low load by stimulating muscle metabolism and hormone secretion, and at the same time reduce joint pressure (22). For older adults people with weak physical functions and those who have had injuries before, since they cannot bear too much burden, blood flow restriction training is the most effective and relatively safe exercise, which can achieve the purpose of exercise without increasing the burden on the joints. In addition, according to individual differences and their exercise needs, the methods and intensities of strength training need to be differentiated. Moreover, in a study on sarcopenia and frailty, it was found during treatment that comprehensive training combining multiple forms of exercise is more beneficial to improving muscle mass and muscle function in the older adults than strength training alone (24).

Studies show that the greater the training intensity, the better the training effect, especially the improvement of lower limb muscle strength and the improvement of daily physical activity ability is more obvious. Therefore, formulating a reasonable exercise intensity and frequency is an important strategy for implementing strength training (25). Expert consensus points out that the intensity of exercise is often expressed as 1RM (one-repetition maximum), which refers to the maximum force that a certain muscle group of the human body can exert in a single contraction and can be measured by means of dumbbells, elastic bands, bench presses or equipment, etc. (18). It is recommended that frail older adults people start resistance training from a low intensity (20–30%-1RM) and gradually transition to a high intensity (80%-1RM) (26). The frequency of exercise should be determined based on the functional conditions of frail older adults people. It is recommended that frail older adults people undergo resistance training at least twice a week, with 1 to 3 sets each time, and each set repeated 8 to 12 times. The rest time between sets should be adjusted to 0.5 to 1 min according to the load, and the number of repetitions should be appropriately reduced as the load increases (7).

### Balanced exercise

Balance training is a training method aimed at restoring or improving the body’s balance ability. It is suitable for patients with poor balance ability caused by central nervous system diseases and the older adults. Balance training stimulates the neuromuscular system, proprioception and vestibular function through specific exercises, enhancing a person’s control over the body. Thus, it can effectively strengthen the core muscle groups of the body, increase joint flexibility, improve body coordination, and significantly improve their balance and motor abilities. Appropriate balance training can effectively reduce the occurrence of falls among middle-aged and older adults people and improve their ability of activities of daily living (25, 27). A study shows that dynamic balance training has a significant effect on improving gait stability and reducing the risk of falls in the older adults (28). Balance training is usually combined with other forms of exercise for multi-dimensional exercise and is more effective in improving the frailty state of the older adults. Studies have found that combining balance training with strength training is more effective in improving the activities of daily living of the older adults and reducing the risk of frailty (17).

Balance training methods are diverse, including single-leg standing, balance board training, Baduanjin, Tai Chi, standing yoga, core strength training, dynamic balance practice, eye-closing training, heel or toe standing (walking on soft surfaces such as foam mattresses), and straight walking, etc. (16, 17). During training, the principle of gradually reducing the supporting area from large to small, from static balance to dynamic balance, gradually increasing the body's center of gravity from low to high, from eye-opening training to eye-closing training, and increasing the difficulty from low to high is adhered to. Some movements (such as specific commands, music or dual-task exercises) can be exercised through eye closure to improve proprioception or cognition. The guidelines recommend conducting balance exercises three times a week, with each training session lasting approximately 10 to 20 minutes, and at least two of them should be carried out under supervision (29). To avoid falls, it is recommended that frail older adults people practice in a safe environment accompanied by caregivers. Timely feedback and adjustment of the exercise plan (18).

### Flexibility exercises

Flexibility training refers to improving the range of motion of joints and the stretching ability of soft tissues such as muscles, tendons and ligaments through systematic training methods, thereby maintaining the flexibility of the body. Studies show that flexibility training can improve the flexibility and coordination of the body. Adhering to flexibility training can also enhance muscle strength, improve body balance function, and reduce the occurrence of falls (30, 31). Flexibility training can also affect a person’s nervous system, improve motor coordination and reactivity, and enhance the quality of life and health level of the older adults. Studies show that for people who persist in exercising for a long time, their neural response speed becomes faster during exercise, their ability to control their own exercise situation is better, and the risk of falls and injuries is also reduced (32, 33). Flexibility training aims to improve physical functions by enhancing physical flexibility, reducing physical stress and improving balance ability. Meanwhile, conducting flexibility exercises before and after exercise can effectively prevent sports injuries. The guideline suggests that the amount of training intensity can be determined based on the patient’s perception. During the exercise, it is advisable to stretch as much as possible until a tight or slightly painful sensation is felt (18). Clinical trial data show that flexibility training can improve the frailty state of the older adults. However, systematic follow-up evaluations and long-term follow-ups are still needed to determine the most suitable flexibility training program and its best effect.

## Evaluation of the effectiveness of physical exercise interventions

Exercise is the main way to improve frailty in the older adults. The evaluation of exercise effects is of vital importance. By assessing the exercise effects, the improvement of physical functions can be understood in a timely manner, and the exercise management plan can be adjusted when necessary. At present, there are various tools for evaluating the effect of exercise internationally, and they are widely used. When evaluating, appropriate assessment tools are selected according to the patient’s exercise plan to effectively assess the effect of their exercise intervention. According to their functions, applications and evaluation methods, exercise effect assessment tools can be classified into scale-based tools, observation-based tools and comprehensive assessment tools. Different assessment tools have different focuses. In recent years, researchers have made remarkable progress in the development and application of exercise effect evaluation tools for older adults frail patients. However, there are still problems such as differences in the effectiveness and applicability among different evaluation tools. Therefore, when conducting evaluations, effective evaluation tools should be selected based on the exercise plans of the older adults in order to better understand their physical conditions.

### Scale-type tools

#### Activities of daily living (ADL) assessment scale

In 1963, the ADL scale was first proposed by Katz et al. in 1969, American scholars Lawton and Brody developed the ADL assessment scale, namely the Activities of Daily Living Scale, to evaluate the activities of daily living ability of the subjects. After continuous development and improvement, it has formed the current ADL scale. ADL is usually divided into Basic activities of daily living (BADL) and Instrumental activities of daily living (IADL) (34). BADL refers to the basic self-care activities necessary for maintaining survival and life, including six aspects: eating, dressing, grooming, using the toilet, walking and bathing. IADL refers to the more complex and demanding daily activities carried out to maintain an individual’s independent life, including eight aspects such as making phone calls, shopping, doing housework, doing laundry, managing medication, taking walks, managing finances and using transportation. These activities usually require the use of tools and involve more cognitive functions. The ADL scale consists of 14 items, with a total score ranging from 14 to 56. A score of ≤ 14 is considered completely normal. A score of 15 to 22 indicates varying degrees of functional impairment, and a score of 23 to 56 indicates significant functional impairment. The lower the score, the better the physical function; conversely, the lower the score, the worse the physical function. According to this scale, the self-care ability and daily activity ability of the evaluated person can be obtained, which can roughly reflect the physical functional state of the evaluated person. It is widely used in clinical practice, has high reliability and validity, and is simple to operate and easy to master, especially suitable for the older adults population.

#### Short physical performance battery (SPPB)

SPPB (short physical performance battery) was proposed by Guralnik et al. in the 1990s, with the aim of conducting a comprehensive assessment of the physical performance of the older adults through a series of standardized tests, including balance, walking speed and repeated sitting up movements. It can well reflect an individual’s physical function status. And with continuous development, SPPB has also been used as a standard for evaluating the physical function and motor ability of the older adults (35). Meanwhile, the score of SPPB is correlated with the quality of life of the older adults. Studies have shown that an increase in the SPPB score can effectively reduce the risk of falls in the older adults, enhance their independence in daily living activities, and thereby significantly improve their quality of life (36, 37). Furthermore, studies have shown that SPPB can not only assess the physical ability of the older adults, but also predict health risks related to the older adults, such as adverse events like falls, hospitalizations and deaths (38, 39). Balance tests are generally carried out in three steps, with the difficulty coefficient increasing step by step. The first step is for the subject to stand with feet together and be able to stand for 10 s (1 point). The second step is to stand with feet half together (the heel of one foot touching the big toe of the other foot) and be able to stand for 10 s (1 point). The third step is to stand with feet together (both feet fully touching front and back) and be able to stand for 10 s (2 points). The score is calculated based on the completion status and the score of physical stability, with a maximum of 4 points. The judgment method of gait speed test is to measure the time it takes for the person being tested to walk a distance of four meters, and classify it according to speed. If it is ≤ 4.82 s, 4 points will be obtained. If it is greater than 8.7 s, 1 point will be awarded. The seat-up test examines the time it takes for the subjects to stand up from a sitting position, reflecting the strength and endurance of the lower limbs of the human body. If the time taken to stand up continuously for 5 times is no more than 11.1 s, 4 points will be awarded. A score greater than 60 s is 0. The total score of this scale is 12 points. Each item is scored from 0 to 4 points. A total score of 10 to 12 points is considered normal, 7 to 9 points is considered mild limitation, 4 to 6 points is considered moderate limitation, and ≤3 points is considered severe limitation. The higher the score, the better the physical function of the subjects; the lower the score, the greater the risk of falls, disabilities and death.

#### Exercise self-efficacy scale (ESS)

This scale was developed by Bandura and translated into Chinese by Tung et al. (40). It refers to an assessment tool for self-behavior awareness and is used to measure an individual’s subjective confidence level in their motor ability and completion of movements (41). ESES consists of 18 items and is scored on a 100-point scale with intervals of 10 points. Each item is scored from 0 to 100 points, with a score of 0 indicating “completely lacking confidence” and being completely unable to engage in physical activities. A score of 100 indicates “complete confidence” and the ability to persist in physical activities. The higher the score, the stronger the confidence in exercise and the greater the possibility that the subject can persist. The scale score is the average of all items. The Cronbach’s *α* coefficient of this scale is 0.96, indicating good reliability.

### Observation tools

#### Berg balance test

The Berg Balance Test Scale was designed by Kathy Berg, a physical therapist from Canada. It consists of 14 items and is scored on a scale of 0 to 4 (42). Stand up from a sitting position, stand without support, and sit without a backrest, respectively. The assessment consists of 14 items, including standing with both feet on the ground or on a stool, sitting from a standing position, transferring, standing with eyes closed without support, standing with feet together without support, extending and moving the upper limbs forward while standing, turning around to look backward while standing, turning 360 degrees, standing with one foot on a step or stool without support, standing with one foot in front without support, and standing on one leg. The total score range of this test is 0 to 56 points, with each assessment item worth 0 to 4 points. A score of 0 indicates that the item cannot be completed or requires significant assistance to complete, while a score of 4 indicates that the inspected item can be independently completed. The scoring criteria include the independence, stability of the action completion, and whether assistance is needed, etc. A score of 0 to 20 indicates poor balance function. A score of 21 to 40 indicates a certain balance ability but there is a risk of falling. A score of 41 to 56 indicates good balance function. A score below 40 indicates a risk of falling. This method has good repeatability tests and inter-group reliability, and has a good correlation with other balance and movement test methods (43). Shumway-Cook et al. once reported that the Berg balance test is the best individual risk indicator of falls for older adults people without neurological diseases and living in the community.

#### Six-minute walking test (6MWT)

The 6-min walk test (6-MWT) was developed by Bakle in the 1960s. It assesses the functional status and reserves of an organism by measuring the distance walked within a specified time. In 1983, scholars such as Butland first used the 6MWT to assess the cardiopulmonary function of patients, and later it was further promoted in the field of cardiac rehabilitation. 6MWT is easy to operate, has good practicability, and the results are reliable and effective. Patients have good tolerance and are easy to accept. It has been widely used in the assessment of clinical functional status and the evaluation of medical intervention effects, etc. The subjects were required to walk back and forth along the corridor as fast as possible within 6 min to test the longest distance they could walk within 6 min. The range of a normal adult’s 6-min walk is 400 to 700 meters. A shortened distance indicates decreased cardiopulmonary function or decreased musculoskeletal function (44). Narotzki et al. (45) found that after 12 weeks of moderate-intensity walking exercise (30 min/d) in older adults healthy subjects, the improvement of 6MWD was the MCID for the improvement of cardiopulmonary disease status. This test is often used to reflect cardiopulmonary function, musculoskeletal status and overall functional reserve.

#### Grip strength measurement

Grip strength refers to the strength of the equal-length contracted muscles in the hand, which can reflect the impact of aging on the physical functions of the older adults. Studies have found that grip strength can not only reflect the functional state of the upper limbs of the older adults, but also the muscle strength of the whole body of the older adults (46–48). The subjects first determined the dominant hand through the left and right hand pre-test and adjusted the grip distance of the dynamometer. Then, they kept their bodies upright, with their feet apart and the upper limbs naturally hanging down. They used the dynamometer to quickly exert force, fully grasping the inner and outer pedals to the maximum extent and maintaining it for 5 s. During the test, grip strength was measured three times repeatedly. There was a 30-s rest between each measurement, and the average value of the three measurements was taken. If a man’s grip strength is less than 26 kilograms and a woman’s is less than 18 kilograms, it is considered low muscle strength. Research (49) indicates that grip strength level can directly affect the daily activity ability of patients, and for every 10 kgf increase in grip strength level, the 6MWD of patients approximately increases by 14 m (50). Therefore, measuring the grip strength of patients can reflect their activity ability level from the side.

### Comprehensive utility tool

#### Comprehensive geriatric assessment (CGA)

The concept of CGA began in 1930, originated in the United Kingdom and developed in the United States. The first CGA scale was the older American resources and services (OARS) scale created in 1975 in the United States. In the following more than 20 years, various assessment tools were created. CGA is also known as comprehensive functional assessment (CFA) for the older adults and multidimensional functional Evaluation of the comprehensive health of the older adults assessment (MFA) is a special multi-level and multidisciplinary assessment and intervention process or model for older adults patients, used to evaluate the physical health, functional status, mental health and social environment of the older adults. As a comprehensive assessment tool, it aims to adopt multi-dimensional assessment methods, Conduct a comprehensive health status assessment of the older adults (51). The important assessment contents of CGA include medical (medical history, medication history, nutritional status and geriatric syndrome, etc.), functional, cognitive and emotional, socio-economic and environmental aspects. Through a comprehensive assessment of the physical condition of the older adults, a treatment plan for the older adults is formulated to improve the quality of life of the older adults. At present, there are various assessment tools for CGA. Such as the comprehensive assessment and referral evaluation (CARE), and the multilevel questionnaire of the Philadelphia geriatric center assessment instruments such as PGCMAI and SF-36 health-related quality of life measure (SF-36 HRQL), etc., all these scales have good reliability and validity. Studies have pointed out that CGA has demonstrated significant clinical value in evaluating the nutritional status, cognitive function and activities of daily living of older adults patients (52, 53). Although CGA is applicable to the assessment of most older adults people, the sensitivity and negative predictive indicators of each screening tool vary depending on the population. Therefore, certain standards and procedures must be followed during the implementation process to ensure the reliability and validity of the assessment results.

## Theoretical aspects of sports management

**Figure 2:**
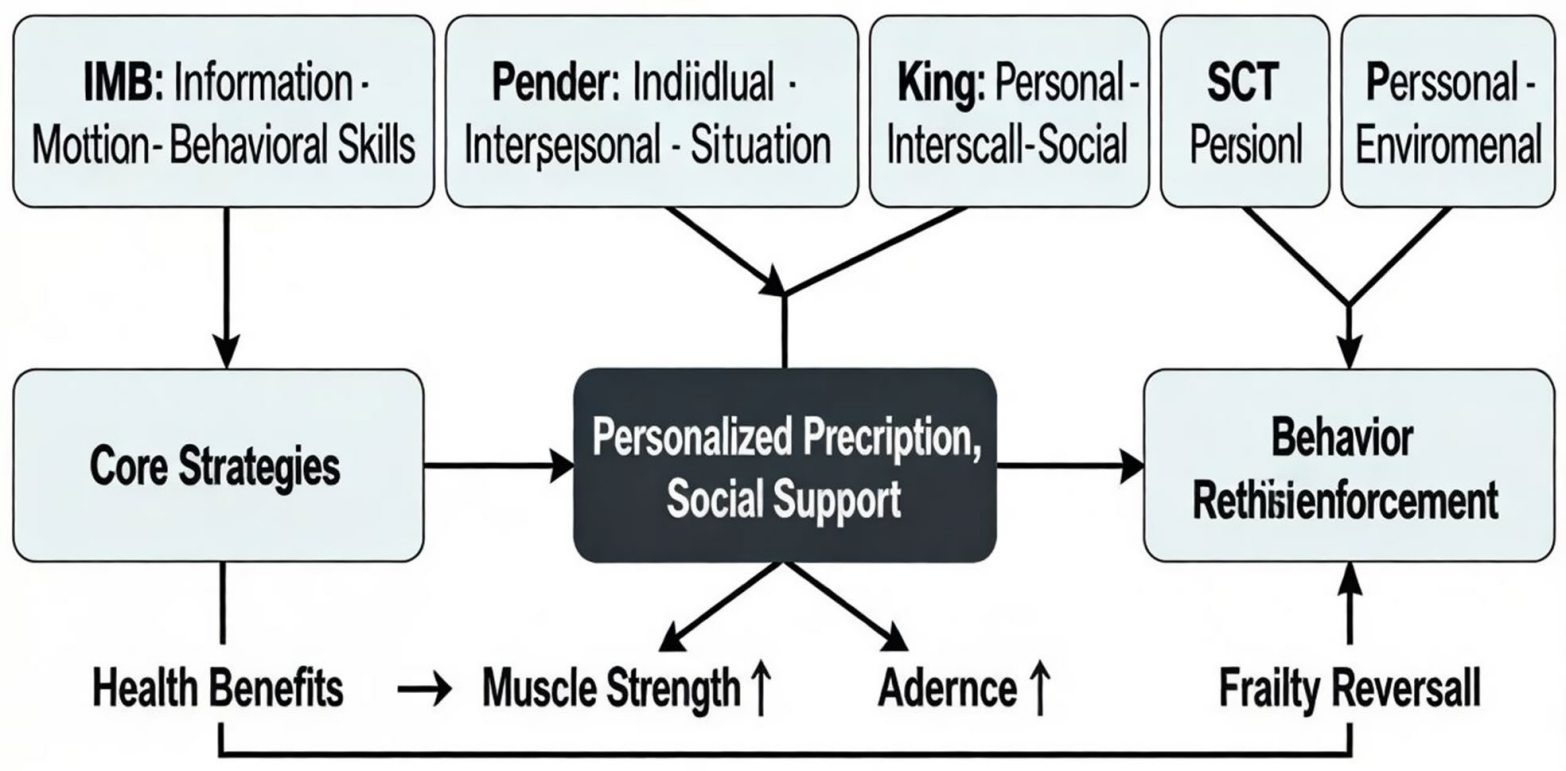
Theory-driven approach to managing frail older adults.

Currently, theories for chronic disease and exercise management are increasingly prevalent internationally. Comprehensive management is achieved through personalized plans, social support, and behavioral reinforcement to attain better exercise management goals. The management pathway is illustrated in Figure 2.

### Information, motivation, behavioral skills model (IMB)

IMB originated from the conceptualized study by Fisher et al. in 1992 on the psychological determinants of risky or evasive behaviors in AIDS. The IMB skills model conceptualizes the psychological determinants that can undermine or promote healthy behavioral performance (54), and critically integrates the structured theories of social psychology and health psychology. The IMB model holds that the relevant information, motivation and behavioral skills of healthy behaviors are the fundamental determinants contributing to healthy behaviors (55). If an individual acquires sufficient information, has a strong motivation to take healthy actions and possesses relevant behavioral skills, they will initiate behavioral changes, maintain healthy behaviors and achieve positive health outcomes. On the contrary, if an individual lacks relevant information, motivation for action and necessary behavioral skills, it will trigger behaviors that are harmful to their health. Information in IBM emphasizes the importance of individuals obtaining accurate, reliable and easily understandable information; motivation focuses on the changes in an individual’s attitude toward healthy behaviors and behavioral norms; behavioral skills focus on providing individuals with the skills necessary to perform preventive behaviors. At present, the IBM model has been widely applied in the field of chronic disease management and behavioral intervention. Its application in the exercise management of older adults frail patients has also received increasing attention in recent years. The results of a multicenter, randomized controlled trial showed that the application of IBM theory could effectively improve the physical function and quality of life of older adults frail patients (56), and another study further verified the practical effect of IBM theory in exercise intervention (57).

### Pender’s health promotion model

Nola J. Pender proposed the health promotion model in 1982, which is mainly used to explain the dynamics of human behavior and the decision-making process of behavior, as well as the behavioral patterns related to health. Its basic idea is that human health behavior is subjective and autonomous behavior. Health promotion is not a single negative defense against diseases. Rather, it is an active behavior in pursuit of health (58). The Nodel J. ender Health Belief Model points out that the important factors influencing health behaviors include four: personal factors; interpersonal factors Situational factors Behavioral intention. Personal factors include an individual’s characteristics and experiences, as well as their specific cognition and emotions. Relevant factors in the physiological, psychological, and social aspects of an individual’s health behaviors can influence their cognition and attitude toward health-promoting behaviors and stimulate their demand for health. Interpersonal factors come from family, peers, communities, etc. Positive interpersonal relationships can promote healthy behaviors. Situational factors refer to the environment for healthy activities. A good environment can induce an individual’s intention for healthy behaviors. Behavioral intention includes two outcomes: the commitment to the action plan and the promotion of healthy behaviors, from which positive health outcomes can be directly obtained. This model provides a scientific framework for health intervention and has been widely applied in chronic disease management. Medical staff design personalized intervention plans by evaluating patients’ health beliefs to promote patients’ healthy behaviors (59). Pender’s model emphasizes people’s initiative and self-management ability in the process of health promotion, and is particularly suitable for the special group of the older adults. Research indicates that when Pender’s theory is applied to the management of frail older adults people, both the physical functions and psychological states of the participants have significantly improved (60); another study shows that under the intervention guided by Pender’s theory, the exercise participation and quality of life of older adults frail patients have been improved (61). These cases not only demonstrate the practical application effect of the Pender model, but also provide valuable practical experience for future research, emphasizing the importance of personalized intervention for older adults frail patients.

### Theory of goal attainment

Founded by American nursing expert Imojenny M. King in 1981, it is influenced by systems theory, symbolic interaction theory and multidisciplinary paradigms. Based on the philosophy that humans are open systems, it has developed from dynamic interaction systems and abstract concepts such as humans, health, environment and society, aiming to provide a scientific framework for individuals in terms of exercise and health management (62). The King attainment theory focuses on elaborating the interactions that occur among people, especially between nurses and patients. The theoretical framework proposed includes: Personal system, interpersonal system and social system. This framework emphasizes that both parties, based on a correct understanding of themselves, their respective roles, and growth and development, achieve mutual influence through effective communication and interaction, and thereby reach the set goals (63, 64). The individual system points out that each individual is an individual system, including sensation, self, growth and development, body image, space, time, etc. Interpersonal system selection indicates that the systems formed by the interaction among people include interaction, communication, mutual influence, roles, emergency response, etc. The social system is a system composed of individuals and groups with mutual relationships in society, including organization, authority, power, status and decision-making. The King attainment theory is based on individualized and comprehensive exercise intervention to improve the functional status and quality of life of older adults patients. Its application in the exercise management of older adults frail patients mainly involves formulating and implementing personalized exercise programs. In recent years, the King attainment theory has been increasingly applied to the exercise management of older adults frail patients, providing a new idea for the quality of life and exercise ability of the older adults (7, 65). The research found that the anti-exhaustion program guided by the King attainment theory can effectively increase the grip strength and walking speed of the older adults (66). In addition, another study found that the King theory of exercise combined with nutrition can effectively improve the physical function and muscle strength of the older adults (67).

### Social cognitive theory (SCT)

Social Cognitive Theory (SCT) is one of the psychological theories proposed by Albert Bandura. Its main viewpoint is that individual behavior is the result of the interaction among individual factors, environmental factors and behavioral outcomes (68, 69). Its three main constituent factors include: Individual factors refer to self-efficacy, emotional state, cognitive ability and individual physiological conditions, etc. Environmental factors refer to the degree of mutual support among friends, family members, community members, the intensity of social support, and the provision of local resources such as families and communities, etc. Behavioral factors refer to a series of behavioral manifestations or coping styles exhibited by an individual in a specific situation (70, 71). Based on this, in the exercise management of older adults frail patients, the aid of social cognitive theory is helpful to explain the causes and characteristics of the exercise behaviors of the older adults and promote their in-depth analysis of the key factors influencing their own exercise behaviors. For example: Research in Finland shows that the multi-faceted intervention methods adopted under the guidance of SCT (such as nutritional guidance, physical exercise, and social activities) are beneficial to the frailty state of the older adults (72). For instance, Miao (73) found in her research that the social activity level of the older adults is closely related to their own health conditions. Excellent social support helps promote the exercise behavior of the older adults and delay the process of weakness. Especially for exercise intervention, the core concept of SCT is conducive to the formulation of exercise management strategies. Strengthen the physical functions of older adults patients through exercise and improve their quality of life.

## Conclusion

At present, research on the older adults in China started later than that in foreign countries, and most of it still remains at the theoretical research stage. With the global aging process, frailty in the older adults has become an important factor affecting the health of the older adults. Exercise management has achieved remarkable results in improving the frailty state of the older adults and is currently an important way to enhance the health level of older adults frailty patients. Despite substantial research supporting exercise as a positive intervention for frailty in older adults, many studies remain flawed. For instance, sample representativeness is often inadequate: most studies feature small sample sizes and predominantly recruit participants from community settings or nursing homes, lacking representative samples from diverse environments such as hospitals or home-bound settings. This limitation restricts the generalizability of findings. Low standardization of intervention protocols: Parameters such as exercise type, intensity, and frequency vary significantly across studies, lacking uniform intervention protocols that hinder systematic comparisons and meta-analyses. Inconsistent assessment tools: Although scales like SPPB and ADL are widely used, modifications or simplifications during application across studies compromise result comparability. Additionally, reliance on self-reported data in some studies introduces potential recall bias or social desirability bias. Lack of long-term follow-up and mechanism studies: Most interventions are short-term (≤6 months), with insufficient systematic exploration of long-term effects, adherence changes, and underlying physiological/psychological mechanisms. Scientific evaluation is crucial for optimizing exercise interventions. While the assessment tools used in this paper are widely applied internationally and domestically, their inconsistent application across studies—often modified or simplified—compromises result comparability. Furthermore, reliance on self-reported data in some studies introduces potential recall bias or social desirability bias. Therefore, in clinical practice, dynamic monitoring should be conducted using assessment tools with appropriate reliability and validity tailored to the characteristics of the subjects to enable precise adjustments to intervention protocols. By evaluating the physical condition of older adults, personalized exercise programs can be developed. Various health management theories provide theoretical guidance for designing targeted exercise programs for frail older adults patients, thereby enhancing the effectiveness of interventions. In summary, while research on exercise management for frail older adults individuals has yielded some achievements, further exploration is needed in standardizing research designs, tracking long-term outcomes, developing personalized interventions, and integrating multidisciplinary approaches. Future efforts should include multicenter, large-sample, long-term randomized controlled trials combined with real-world studies to establish evidence-based exercise management guidelines suitable for diverse cultural and social contexts. This will ultimately advance the scientific and humanized approach to comprehensive health management throughout the frailty cycle in the older adults.
