# Correction: Research advances in exercise management for frail older adults

**DOI:** 10.3389/fpubh.2026.1869748

**Published:** 2026-05-19

**Authors:** Caini Liu, Lan Lu, Wei Wei

**Affiliations:** 1 Nursing College, Guangxi Medical University, Nanning, China; 2 Department of Nursing, First Affiliated Hospital of Guangxi Medical University, Nanning, China

**Keywords:** assessment of aging, frail older people, exercise and health management, research progress, tool

In the published article, there were mistakes in the **Reference** list. Several references were inadvertently duplicated. The corrections are as follows:

7. Yang X, Li S, Xu L, Liu H, Li Y, Song X, et al. Effects of multicomponent exercise on frailty status and physical function in frail older adults: a meta-analysis and systematic review. *Exp Gerontol*. (2024) 197:112604. doi: 10.1016/j.exger.2024.112604

28. Yang X, Li S, Xu L, Liu H, Li Y, Song X, et al. Effects of multicomponent exercise on frailty status and physical function in frail older adults: a meta-analysis and systematic review. *Exp Gerontol*. (2024) 197:112604. doi: 10.1016/j.exger.2024.112604

69. Yang X, Li S, Xu L, Wang Y, Zhang J, Chen H, et al. Effects of multicomponent exercise on frailty status and physical function in frail older adults: a meta-analysis and systematic review. *Exp Gerontol*. (2024) 197:112604. doi: 10.1016/j.exger.2024.112604

17. da Silva Capanema B, Fank F, Machado Trento MC, Lima Costa D, da Amaral Rocha AR, Zarpellon Mazo G. Home-based exercise programs for the oldest-old to attenuate physical frailty: a scoping review. *J Frailty Aging*. (2024) 13:369–83. doi: 10.14283/jfa.2024.41

19. da Silva CB, Fank F, Machado Trento MC, Lima Costa D, da Amaral Rocha AR, Zarpellon Mazo G. Effects of different exercise interventions on falls, gait, and balance in physically frail older adults: a systematic review and meta-analysis. *Exp Gerontol*. (2024) 197:112604. doi: 10.1016/j.exger.2024.112604

32. da Silva Capanema B, Fank F, Machado Trento MC, Lima Costa D, da Amaral Rocha AR, Zarpellon Mazo G. Home-based exercise programs for the oldest-old to attenuate physical frailty: a scoping review. *J Frailty Aging*. (2024) 13:369–83. doi: 10.14283/jfa.2024.41

26. Wang Y, Yang X, Liu H, Feng Q, Li Y, Hou W, et al. Characteristics of frailty phenotype in Chinese nursing home population and significance of motor function indicators in frailty assessment. *Medicine*. (2022) 101:e31971. doi: 10.1097/MD.0000000000031971

30. Wang Y, Yang X, Liu H, Feng Q, Li Y, Hou W, et al. Characteristics of frailty phenotype in Chinese nursing home population and significance of motor function indicators in frailty assessment. *Medicine*. (2022) 101:e31971. doi: 10.1097/MD.0000000000031971

82. Martínez-Montas GF, Sanz MS-M, Benítez-Silero d JD, Martínez-Aranda LM. Prevention and alleviation of frailty syndrome in older adults and middle-aged people through physical activity: a systematic review. *Health Care*. (2025) 3:276. doi: 10.3390/healthcare13030276

84. Martínez-Montas GF, Sanz-Matesanz M, Benítez-Sillero JD, Martínez-Aranda LM. Prevention and mitigation of frailty syndrome in institutionalised older adults through physical activity: a systematic review. *Healthcare*. (2025) 13:276. doi: 10.3390/healthcare13030276

References [7], [28], and [69] were duplicates of the same source. The correct reference is [7]; references [28] and [69] have been deleted.

References [26] and [30] were duplicates of the same source. The correct reference is [26]; reference [30] has been deleted.

References [17], [19], and [32] were duplicates of the same source. The correct reference is [17]; references [19] and [32] have been deleted.

References [82] and [84] were duplicates of the same source. The correct reference is [84]; reference [82] has been deleted.

The original version of this article has been updated.

